# The role of product cues and regulatory focus in the consumers’ response to green products: The mediation effects of green attitudes

**DOI:** 10.3389/fpsyg.2022.918248

**Published:** 2022-10-03

**Authors:** Xiaomei Wang, Yangli Gu, Haohang Xin, Peiling Qiu, Jia Wang

**Affiliations:** School of Media Studies and Humanities, Zhejiang University City College, Hangzhou, Zhejiang, China

**Keywords:** brand strength, retailer reputation, regulatory focus, green attitude, consumers’ response

## Abstract

By applying the cue-diagnosticity theory, this study explores the influence mechanism of consumption response to low-involvement products and high-involvement products, respectively. Specifically, the purpose of this study is to investigate how product clues (brand strength and retailer reputation) affect consumption responses to green products with different involvement and to examine regulatory focus as a moderator and green attitude as a mediator. The results of study 1 reveal that for low-involvement green products, the effect of the retailer reputation rather than brand strength on consumption response is mediated by a green attitude, and the regulatory focus plays a moderating role in this process. The results of study 2 show that for high-involvement green products, the effect of the brand strength rather than retailer reputation on consumption response is mediated by a green attitude; however, the regulatory focus does not play a moderating role in this process. Finally, the data aggregation verifies that people’s consumption response to green products strongly depends on the retailer reputation, brand strength, and green attitude, and there is a moderated mediation effect of regulatory focus on the indirect effect of retailer reputation (rather than brand strength) on consumption response *via* green attitude. As behavioral antecedents differ across the analyzed product types in forming consumer response, it is very important for policymakers and marketers to take note of the differences when designing marketing activities for green products.

## Introduction

Since the 1990s, with the deterioration of the ecological environment, consumers’ awareness of environmental protection and sustainable development has continually increased, and the concept of green consumption has also emerged. With the establishment of an environmental system and the improvement of consumers’ awareness of green consumption, an increasing number of business decision-makers are paying attention to the production and marketing of green products. Enterprises spread the concept of environmental protection to consumers through green marketing and improve consumers’ willingness to buy green products, which is of great significance for establishing brand image and expanding the market share of green products. Consuming green products instead of non-green products not only optimizes the consumption structure but also establishes sustainable consumption patterns. However, to ensure environmental attributes and a sustainable concept, there may be certain disadvantages in terms of price, appearance, and convenience, which somewhat weakens consumers’ purchase intention. Therefore, it has become both an academic and business concern to explore the mechanisms and influence boundaries of consumer intention to purchase green products.

Green products are products that do not harm the environment or contain potentially harmful ingredients. Paul et al. (2016) defined green products as those that did not pollute the earth or destroy natural resources and could be recycled or utilized. Green products do not pollute the environment or damage natural resources and can be recycled or used to save energy. Compared with ordinary products, green products tend to use non-toxic raw materials that can be degraded by microorganisms, use packaging that can be recycled, consume less energy, and cost more than ordinary products. They are new products and have not been widely accepted by society. Therefore, the trade-off of product attributes makes consumers experience psychological conflicts, thus hindering green consumption (Olson, 2013). In the face of the contradictory characteristics of green products, how would different information about green products affect consumers? Who would present a positive consumption response? What is the psychological mechanism of this difference?

Consumers use a variety of available information to form their own attitudes. According to the ABC model of attitudes, attitude consists of three components: affect, behavior, and cognition (Solomon, 2011). Due to the different interactions of the three components, the ABC construct has three attitude models, one of which is based on cognitive information processing. The model holds that consumers first form the cognition of an object by actively collecting relevant information in the process of purchase decision-making. Then, through the comprehensive evaluation of the relevant cognition, consumers develop an emotion toward the object. Finally, consumers form the intention to engage in certain purchase behaviors. Thus, consumers gather much information and carefully weigh the pros and cons. In addition, the determinants of purchase intention in the online environment are somewhat different from those in the offline environment. However, in an online environment, the cost of searching for price and other product information is usually low because there is no shipping cost. However, the lack of storefronts and the inability to try products increase the uncertainty of online consumers. In many cases, online consumers do not know the true quality of the product and do not know how to make an appropriate consumers’ response (Purohit and Srivastava, 2001). As a result, online consumers’ evaluation of products depends on online information clues, such as brand name, retailer reputation, and online reviews. Consumers can reduce this uncertainty by turning to well-known manufacturer and retailer brands (Vijayasarathy and Jones, 2015). As consumers need to combine multiple cues when making judgments, previous literature has used the cue-diagnosticity framework to explain the impact of multiple cues on consumer evaluation. The brand name and retailer reputation develop over time, and the value of such clues is relatively permanent in nature, which have important impacts on consumers’ judgment (Purohit and Srivastava, 2001). But are these two clues (brand strength and retailer’s reputation) consistent in the valence across different product types? However, this problem has not been substantially confirmed in the literature on green consumer behavior. Based on the cue diagnosticity, this research fills this gap by examining the role of brand strength and retailer reputation in the context of products with different involvement.

Although enterprises use various product information cues to promote their products, these cues may interact with consumers’ internal influencing factors (such as motivation and attitude). Researchers have also noted that the input of product information cues and consumers’ cognitive, attitudinal, and behavioral responses are modulated by their motivations (Vakratsas and Ambler, 1999). Therefore, understanding the interplay between product cues and consumer motivations and attitudes is a key ongoing issue for marketers. This is also one of our main research objectives.

The current study aims to build a generalizable model to understand the joint effect of product cues (i.e., brand strength and retailer reputation), green attitudes, and regulatory focus on consumers’ response. Specifically, this current study explores how brand strength and retailer reputation interact with green attitudes and regulatory focus. It is hoped that understanding the factors that affect the consumption response of green products improves the overall effectiveness of green marketing activities. The remainder of the article is organized as follows: we first propose a conceptual framework and present our hypotheses. Secondly, in study 1, we explored the influence of brand strength, retailer reputation, green attitudes, and regulatory focus on the consumption response of green products with low involvement. Next, study 2 tests the predictions by green products with high involvement. Then, the previous two experimental data were summarized and analyzed. Finally, we discuss the theoretical and practical implications of our findings. Limitations and future research avenues conclude the paper.

## Literature review and hypothesis development

### Cue-utilization theory and cue-diagnosticity theory

Cues are signals representing the development context and attributes of things. In a sense, a cue is a collection of information. This kind of clue or collection of clues will influence the judgment and decision-making of individuals. The application of cue theory has attracted increasing attention in academic circles and has accumulated a large amount of valuable research literature.

Cue-utilization theory suggests that consumers usually make a comprehensive analysis of a series of cues transmitted by products and choose what they think is useful as the basis for judging the quality and making purchase decisions (Cox, 1962). Due to the existence of information asymmetry, consumers cannot directly and accurately judge the quality of goods, so they can only infer the quality of goods through cues provided by producers and sellers to reduce the uncertainty in the decision-making process. The cues can be internal and external (Olson and Jacoby, 1972). Internal cues refer to the inherent characteristics of the product itself, including product size, shape, and taste, which do not change and are not controlled by the outside world. External cues refer to the attributes related to products that can be changed, including price, brand name, store reputation, and so on. However, internal cues are difficult to obtain, so consumers tend to use external cues to evaluate the quality of products, especially when shopping online (Rao and Monroe, 1988).

Based on clue utility theory, Purohit and Srivastava (2001) proposed the cue-diagnosticity framework, which posits that an individual’s judgment of product quality takes place by classifying products to a certain quality level by using appropriate cues. The theory proposes that clues can be roughly divided into two types: high-range clues and low-range clues (Gidron et al., 1993). High-range clues (such as brand name and company reputation) are those that evolve over time, and their valence cannot be easily changed. In contrast, low-range clues (such as product price and warranty) are short-lived and can be changed relatively quickly. Compared with low-range clues, high-range clues are considered more credible and, thus, more diagnostic. High-range clues are also be regarded as “independent” clues; in other words, the diagnostic value of such clues is relatively less dependent on the existing value of other clues. Previous studies have demonstrated that there was an interactive effect between the diagnostics of different clues (Purohit and Srivastava, 2001), and when multiple cues coexist, the influence of a lower diagnostic cue on consumption response depends on the valence of a higher diagnostic cue (Wang C. et al., 2022). Different clues might be given different degrees of attention and weight. High-range cues have higher diagnostic value and are more useful for consumers’ evaluation and decision-making (Akdeniz et al., 2013). The main goal of the current research is to explore the difference of the influence mechanism of high-range cues (brand strength and retailer reputation) on consumers’ responses to green products with different involvement.

### The role of brand strength and retailer reputation in consumers’ response

Consumers’ response generally involves two aspects: brand (what brand to buy) and retailer reputation (where to buy). For example, when consumers need a tracksuit, they are faced with the decision of choosing a brand such as Adidas or a lesser-known brand. They also need to decide where to buy, either at the manufacturer’s own store or at a retail outlet. Various combinations of brands and retailers form the basis of consumers’ response. Research in the literature has documented the power of brand and retailer reputation to influence consumer perceptions and purchasing decisions. A brand or retailer with a high reputation has a more positive impact on consumer perceptions and behavior.

In many cases, consumers do not know the true quality of products (or brands) before making purchase decisions. Until consumer behavior is carried out, the quality of the goods cannot be observed. Under these circumstances, research shows that consumers may rely on some signals or clues to evaluate product quality. Brand signal theory suggests that the uncertainty of product quality and performance represent risks to consumers’ purchasing process (Erdem et al., 2006). To avoid risks, consumers will seek as many clues as possible (for example, brand name, price, and warranty) to predict the quality and performance of products (Korfiatis et al., 2012). Mühlbacher et al. (2016) define brand advantage as “an evaluative or behavioral response such as commitment, trust, reputation, or recommendation…that affects brand choice” (p. 2,774). For products with brand advantages, the more information consumers have, the greater the perceived value (Grewal et al., 1998). Thus, a good brand image is an integral part of brand value, which enhances consumers’ perceived value; that is, strong brands have greater credibility than weak brands and transmit reliable product information to consumers through strong brand signals. In our context, we divide brands into those with low brand strength and high brand strength; the former are relatively unknown brands, and consumers are less familiar with them; the latter are well known in their target market, and consumers are more familiar with them; that is, the products have a high level of popularity and reputation.

Reputable retailers also play an important role in many purchasing activities, delivering high-quality images to consumers. Retailers provide communication channels between consumers and manufacturers. A retailer with a good reputation has sufficient motivation to maintain its reputation; then, it is likely to eliminate inferior products and provide high-quality products. Purohit and Srivastava (2001) show that regardless of the reputation of the manufacturer, when the manufacturer sells products through reputable retailers, the perception of product quality is higher. There are relatively abundant studies on the impact of store patronage and retailer attributes. Dabija and Bǎbut (2019) have concluded that retailers’ sustainable behavior, assortment, personnel, and advertising have impacts on Millennials’ store patronage. Similar to offline consumption, if a product is sold through a well-known online retailer, consumers are willing to buy the product. Internet transactions have brought great uncertainty to consumers, but reputable retailers help reduce this uncertainty. That is, retailers with a good reputation on the internet will promote consumer willingness (Chu et al., 2005).

As seen from the above, the reputation of both manufacturers’ brands and retailers is an important clue for consumers. The brand is directly related to the product, and the brand strength is more specific because it is suitable for relatively few products, while the retailer reputation is indirectly related and applies to all kinds of products in the store. Retailers with a high-quality reputation have an incentive to maintain their reputation by screening poor-quality manufacturers and providing high-quality products. To some extent, the manufacturer’s reputation can be used as a tool for product quality signals. A manufacturer without a strong brand may “borrow” the reputation of the retailer to mark quality (Chu and Chu, 1994). Empirical research shows that a strong retailer brand marks high-quality products and leads to higher consumers’ purchase intention. If the product produced by a well-known manufacturer is sold by a well-known retailer, most consumers will evaluate the product more positively (Zhu and Chen, 2017) because product brands and retailer reputations are mortgage bonds with the quality signal. A strong brand can make up for the reputation of a lower retailer, and vice versa; that is, if the product is a weak brand, consumers’ perceived quality of the product will also be improved with a higher retailer reputation (Chu et al., 2005). Therefore, we put forward the following hypothesis:

**H1:** Brand strength and retailer reputation jointly affect consumption response to green products.

### Regulatory focus and product cues

Higgins (1997) first put forward regulatory orientation theory (regulatory focus theory) and held that people tend to move toward a certain type of goal state. There are two main types of regulatory focus: prevention focus and promotion focus. Promotion-focused individuals pursue a perfect and ideal goal, focus on whether they can benefit or not, and adopt an “eager strategy,” while prevention-focused individuals pursue a goal of obligation or responsibility, focus on whether they will lose or not, and adopt a “vigilant strategy” (Cesario et al., 2004). It is found that when a specific regulatory focus matches compatible behavior strategies (such as promotion focus and eager strategy, prevention focus, and vigilant strategy), it will produce “value derived from matching” (Avnet and Higgins, 2006), and the fit will also lead to enhancement effects in many aspects, such as motivation level, behavior quantity, and performance (Avnet and Higgins, 2006).

With the increase in brand familiarity, consumers will develop an emotional connection with the brands, which in turn leads to a stronger willingness to buy familiar brands. That is, familiar brands will make consumers have less uncertainty and risk and give higher recognition to those products. However, for brands with low familiarity, consumers will feel more uncertainty and risk, which will make individuals with a prevention focus (who tend to adopt a “vigilant strategy”) less willing to buy than those with a promotion focus (who tend to adopt an “eager strategy”). For brands with high familiarity, consumers refer more to internal information such as previous experience and product-specific attributes, which leads to no significant difference in purchase intention among consumers with different regulatory focuses (Meng et al., 2019).

One objective of this study is to explore whether consumers with different regulation focuses react differently to product cues (brand strength and retailer reputation). Although ample research has investigated the main effects of product cues, limited research has explored the complexity of the interplay between product cues and regulatory focus. For instance, Song and Morton (2016) discussed the degree of interaction between product cues and individual regulatory focus when evaluating products and found that consumers with a promotion focus thought external cues were more important and had a more favorable evaluation of products with superior external cues. On the other hand, consumers with a prevention focus give more importance to the internal cues of the products, so they will have a more favorable evaluation of the products with higher internal cues. In summary, we put forward the following hypothesis:

**H2:** There is an interactive effect between the regulatory focus and product cues (brand strength and retailer reputation) in the process of influencing green product consumption response.

### The intermediary role of green attitudes

In the field of consumption, an attitude refers to a person’s judgment of products and services, and attitude is an important antecedent of consumer behavior intention (Dhir et al., 2021). Attitudes are important influencing factors of green consumption. When a person has a positive attitude toward the environment, he or she will pay more attention to environmental issues, which may prompt him or her to replace non-green products with green products (Cheung and To, 2019). A positive attitude toward the purchase of green products can be regarded as the starting point of sustainable consumption, which can lead to positive consumers’ responses to green products. Individual attitude plays a major role in forming purchase intention, followed by subjective standards and perceived behavioral control (Scalco et al., 2017; Lǎzǎroiu et al., 2019). Kumar et al. (2017) suggested that consumers with attitudes toward environmental issues are more likely to buy green products. When consumers know that concern for the environment is more beneficial to the society, they will buy more green products. Green attitude is an intermediary variable between individual altruistic values and environmentally friendly behaviors (Lǎzǎroiu et al., 2019). The scholars Han and Kim (2010) also believed that people’s green attitudes strengthened their intention of consuming certain products. Dhir et al. (2021) pointed out that consumers’ attitudes would affect the consumer behavior intention toward green products, but there was a significant gap between attitude and behavior. Compared with ordinary consumption behavior, the decision-making process of green consumption is extremely complex. From product evaluation to product purchase, the conflict between personal interests and social interests and between current interests and long-term interests is a constant. However, consumer attitudes are important predictors of consumer behavioral intention toward green products. Riskos et al. (2021) emphasized that the attitude toward green products was an important intermediary variable of purchasing behavior. Consumers’ green product attitude plays an intermediary role in the influence of green product literacy, green product orientation, social influence, and green customer value on behavior intention (Chen et al., 2022; Wang Y. M. et al., 2022). Based on this fact, we propose the following hypothesis:

**H3:** Green attitudes play an intermediary role in the response of product cues (brand strength and retailer reputation) to green product consumption.

### Product involvement

Involvement refers to a state of motivation that is related to the needs and values of consumers (Rothschild, 1984). Product involvement is consumers’ perception of the importance and value of products based on their inherent interests, concepts, and needs (Xuemei and Luiz, 2011). For low-involvement products, consumers spend less time and search behavior to make correct and effective purchase decisions, and they tend to spend relatively less time and energy on product information collection and collation and follow the principle of making the least effort to make consumption decisions. However, for highly involved products, consumers need to spend much time and search behavior to ensure that they make wise purchase decisions, they have a strong motivation to deal with product information, and they will be more willing to process each piece of product information carefully and systematically. Moreover, they will be more attentive when viewing product information (Kim and Han, 2014). To put it simply, consumers with a high level of involvement tend to search for more product information to compare and evaluate products, whereas consumers with low involvement tend to ignore the steps of searching for information and making direct purchases. Rahman (2018) highlighted that consumers tend to make different judgments about the information related to green products according to the degree of involvement of the products, and then there are differences in consumption decisions. Dabija et al. (2018) found that in the Romanian retail market, the behavioral antecedents of retail formats in building green loyalty were different. Based on the above literature, we argue that the behavioral antecedents of consumption reactions might be different for green products with different involvement. Therefore, this manuscript contains two surveys about products with different involvement: study 1 focuses on low-involvement green products (green laundry detergent), and study 2 focuses on high-involvement green products (environmentally friendly mobile phones).

## Study 1

### Method

#### Participants and procedure

A total of 117 undergraduates participated in this study for course credit. The participants were randomly assigned to one of the four cells of a 2 (brand strength: high and low) × 2 (retailer reputation: good and poor) between-subjects design.

The participants were told that they would be participating in a series of research studies. More specifically, the first study included the priming manipulations of retailer reputation and brand strength by asking the subjects to read some materials. Some examples of experimental materials included the following: “You want to buy eco-friendly phosphate-free laundry detergent online; phosphate-free laundry detergent can effectively reduce the discharge of phosphorus-containing sewage and prevent water quality from deteriorating. You found a brand named DARAS, which was one of the emerging brands in the laundry and care industry. Its products had just entered the laundry and care market, and its market share is relatively low.” After reading the materials, they completed a series of self-administered questionnaires, which included questions related to the variables, manipulation checks, and demographic questions. The entire process took approximately 20 min.

#### The measurement of the constructs

All items were measured using a 7-point Likert scale from 1 (very low/bad/strongly disagree) to 7 (very high/good/strongly agree). This research required each respondent to identify specific information and green products of a given brand to indicate which gave them the best experience. Next, each respondent was asked to focus on the questionnaires relating to the given brand. The purpose of the first part of the questionnaire was a manipulation test of the independent variables, and the second part was used to measure the dependent, moderating, and mediating variables. We reported more information on the questionnaires in Appendix A and described the measurement of this study in the following.

##### Brand strength and retailer reputation

The measure of brand strength was adopted from Xiangdong (2017), including two items, e.g., “What do you think about the brand strength of the brand? It was a 7-Likert scale (from 1 = very low to 7 = very high) with high scores indicating high brand strength. The measurement of retailer reputation was developed by Chao (2016) and Purohit and Srivastava (2001) with three items, e.g., “The retailer has a high overall reputation score.” It was a 7-Likert scale (from 1 = strongly disagree to 7 = strongly agree) with high scores indicating good retailer reputation. The Cronbach’s alpha coefficients are 0.891 for brand strength and 0.879 for retailer reputation.

##### Green attitude

A four-item measure was provided by Al-Swidi and Saleh (2021). The questions were anchored on a 7-point scale (from 1 = strongly disagree to 7 = strongly agree). Participants were asked to indicate their opinion on each of the questions, e.g., “I prefer green products because they are environment friendly.” The Cronbach’s alpha for this scale was 0.867.

##### Regulatory focus

The scale of regulatory focus was developed by Higgins (1997) and Bai-Lin (2015). Participants indicated their level of agreement with the eight items on 7-point Likert scale. Four of these items measure promotion focus, e.g., “I have been striving to fulfill my hopes and aspirations.” The remaining four items measure prevention focus, e.g., “I work hard to prevent failure and falling behind.” The Cronbach’s alpha for this scale was 0.823.

##### Consumers’ response

A six-item measure was adopted from Yong (2020). Half of the items measured emotional response, e.g., “I am eager to learn more information of this brand related to environmental protection,” and the remaining half described behavioral response, e.g., “I will recommend the brand to others.” The Cronbach’s alpha for this scale was 0.884.

### Results

#### Effects of brand strength and retailer reputation on emotional response

An analysis of variance was performed. The results showed that in low-involvement products, brand strength had no significant response to consumer emotions. [*F*(1,113) = 1.80, *p* = 1.18]. However, retailer reputation had a significant marginal response to consumers’ emotion [*F*(1,113) = 3.46, *p* = 0.065], and the interaction effect between brand strength and retailer reputation is not significant [*F*(1,113) = 0.04, *p* = 0.84]. For products with low involvement, consumers are more concerned about the reputation of retailers than brand strength. Therefore, manufacturers of enterprises should choose retail channels with better reputations to arouse the better emotional identities of consumers. Thus, we concluded that the emotional response of consumers is more influenced by retail reputation than by manufacturing brands. It also followed that enterprise manufacturers should choose retail channels with better reputations to arouse better emotional recognition of consumers.

#### Effects of brand strength and retailer reputation on behavior response

Through the analysis of variance, we found that for low-involvement green products, brand strength [*F*(1,113) = 9.00, *p* = 0.003] and retailer reputation [*F*(1,113) = 8.30, *p* = 0.005] have significant effects on behavior response, but their interaction effect is not significant [*F*(1,113) = 1.36, *p* = 0.25]. Specifically, the behavioral response in the high brand strength condition was higher than in the low one, and the behavioral response in the poor retailer reputation condition was lower than in the good one. In this way, for green products with low involvement, there were two independent ways to arouse consumers’ positive behavior response: one was to create a good brand image, and the other was to choose retailers with a good reputation. This was consistent with the conclusion of previous studies that both brand strength and retailer reputation belong to high-range clues; that is, the diagnostic value of these high-range clues was relatively less dependent on the value of other clues, and they would independently affect consumers (Song and Morton, 2016).

However, according to previous data, it is known that for low-involvement green products, brand strength has no significant response to emotion. We proposed that before buying low-involvement green products, consumers often do not have much involvement in the brand of the product, and they do not want to know much about the product information related to green environmental protection. However, when consumers make the decision to buy green products with low involvement, that is, when purchasing decision-making behavior occurs, the main effects of brand strength and retailer reputation are significant. We speculated that consumers would not collect too much product information beforehand when purchasing low-involvement green products, tending to rely on consumption experience or habits, and were loyal to a certain brand or a retailer with a high reputation (Jian et al., 2020).

#### Effects of brand strength, retailer reputation, and regulatory focus on consumer response

The analysis of variance showed that the interactive effect of brand strength, retailer reputation, and regulatory focus on emotional response is insignificant [*F*(1,109) = 2.048, *p* = 0.155, $\eta_{p}^{2}=0.018$], while the interactive effect of retailer reputation and regulatory focus on emotional response is marginal [*F*(1,109) = 3.454, *p* = 0.066, $\eta_{p}^{2}=0.031$]. Further simple effect analysis found that under the condition of poor retailer reputation, the emotional responses of promotion-focused individuals (*M* = 4.527, SD = 0.275) are slightly higher than those of prevention-focused individuals (*M* = 3.900, SD = 0.246) (*p* = 0.092). However, for the prevention-focused individuals, the emotional response under the condition of good retailer reputation (*M* = 4.768, SD = 0.275) is significantly higher than under the condition of poor retailer reputation (*M* = 3.900, SD = 0.246) (*p* = 0.010). Based on these findings, we suggested that when retailer reputation is relatively poor, it was possible to start the promotion focus of consumers through information frameworks of “acquisition,” and then consumers are stimulated to have a higher emotional response to the product. In addition, for individuals with prevention orientation, good retailer reputation has high emotional attractiveness, so if the products are low involved, it is necessary to strengthen the construction of retailer reputation.

The analysis of variance showed that the main effect of retailer reputation [*F*(1,109) = 7.922, *p* = 0.006, $\eta_{p}^{2}=0.068$] and the main effect of brand strength [*F*(1,109) = 8.680, *p* = 0.004, $\eta_{p}^{2}=0.074$] on the behavior response are significant, and the interactive effects of brand strength, retailer reputation, and regulatory focus on behavior response were marginal [*F*(1,109) = 3.366, *p* = 0.069, $\eta_{p}^{2}=0.030$].

To further explore the influence of brand strength, retailer reputation, and regulatory focus on emotional response, we made *post hoc* multiple comparisons (LSD). Groups 1–8 are 1 = promotion focus, low brand strength, and poor retailer reputation; 2 = promotion focus, high brand strength, and poor retailer reputation; 3 = promotion focus, low brand strength, and good retailer reputation; 4 = promotion focus, high brand strength, and good retailer reputation; 5 = prevention focus, low brand strength, and poor retailer reputation; 6 = prevention focus, high brand strength, and poor retailer reputation; 7 = prevention focus, low brand strength, and good retailer reputation; and 8 = prevention focus, high brand strength, and good retailer reputation. The main results are as follows.

(1) The difference in emotional response between Group 1 and Group 5 was marginally significant (*p* = 0.07); that is, under the condition of low brand strength and poor retailer reputation, promotion-focused individuals tended to have a more positive emotional response to green products than prevention-focused individuals.
(2) The emotional response of Group 4 was significantly different from that of Group 5 (*p* = 0.005) and Group 6 (*p* = 0.054). That is, under the product clue of high brand strength and good retailer reputation, the emotional responses of the promotion-focused individuals were higher than those of the prevention-focused individuals under the low (or high) brand and low retailer reputation. For individuals with a prevention focus, regardless of whether the brand was strong or weak, if retailer reputation was poor, their emotional impact on products was significantly lower.
(3) The difference in emotional response between Group 5 and Group 8 reached a significant level (*p* = 0.010). That is, compared with product clues with low brand strength and poor retailer reputation, individuals with a prevention focus tended to have more positive emotional responses to high brand strength and good retailer reputation. Correspondingly, there was no significant difference between Group 1 and Group 4 (*p* = 0.417). Therefore, we can infer that prevention-focused individuals pay more attention to brand strength and retailer reputation than promotion-focused individuals.
(4) The emotional response of Group 5 and Group 7 (*p* = 0.086) (vs. Group 6) was marginal (not significant, *p* = 0.278). That is, compared with product clues with a low brand and poor retailer reputation, individuals with a prevention focus tended to have a more positive emotional response to green productions with a low brand and good retailer reputation but no more positive emotional response to those with a high brand and poor retailer reputation. Again, it was proven that prevention-focused individuals pay more attention to the product clues related to retailer reputation, and when retailers have a good reputation, their emotional response is high.

Similarly, we also explored the influence of brand strength, retailer reputation, and regulatory focus on behavior response and made *post hoc* multiple comparisons (LSD). Groups 1–8 are 1 = promotion focus, low brand strength, and poor retailer reputation; 2 = promotion focus, high brand strength, and poor retailer reputation; 3 = promotion focus, low brand strength, and good retailer reputation; 4 = promotion focus, high brand strength, and good retailer reputation; 5 = prevention focus, low brand strength, and poor retailer reputation; 6 = prevention focus, high brand strength, and poor retailer reputation; 7 = prevention focus, low brand strength, and good retailer reputation; and 8 = prevention focus, high brand strength, and good retailer reputation. The results are as follows.

(1) The behavioral responses of Group 4 (vs. Group 8) were significantly higher than those of Groups 1 and 5. The behavioral responses of high brand strength and good retail reputation were significantly higher than those of low brand and low poor reputation. The behavioral response of Group 4 was significantly higher than that of Groups 2 (*p* = 0.014) and 3 (*p* = 0.013). Green products with low involvement, high brand strength, and retailer reputation have a very significant impact on the behavioral response of individuals with a promotion focus.
(2) It is worth noting that the differences in behavioral responses between Group 1 and Group 5 were marginal (*p* = 0.082). Both groups were under the condition of low brand strength and poor retailer reputation, which showed that the promotion-focused individuals tended to have more positive behavioral responses to green products than those with a prevention focus. That is, when the brand strength of green products was not high and the reputation of retailers was not good enough, starting consumers’ promotion focus through marketing strategies may promote their behavioral responses.
(3) There were significant differences in behavioral responses between Group 5 and Group 6 (vs. Group 7); that is, under the low strength and bad reputation conditions, it had a negative response to the behavioral response of prevention-focused individuals. Therefore, for individuals with a prevention focus, merchants and marketers need to strive to improve the brand image of products or choose retailers with higher reputations to obtain a better consumer behavior response. However, there was no difference between Group 1 and Group 2 (vs. Group 3) in the promotion-focused groups, which were the same combinations of brand strength and retailer reputation as Groups 5, 6, and 7. Therefore, we believe that when the brand strength and retailer reputation were low or one of the clues was low, the difference in the behavioral response of individuals with a promotion focus was not significant.
(4) The behavioral responses of Group 8 and Groups 1, 2, 3, 5, and 6 were significant. However, it is worth noting that there was no significant difference between Group 8 and Group 7; that is, for individuals with a prevention focus, even if the brand strength of green products with low involvement was not high, they would still receive a more positive response under the condition of a high retailer reputation. From this, it can be inferred that the quality of retail reputation was very important for green products with low involvement, especially for prevention-oriented individuals.

#### The mediating effect of green attitude between brand strength (vs. retailer reputation) and consumers’ response

The results of mediator analysis with selected retailer reputation as the independent variable, green attitude as the mediating variable, and emotional response as the dependent variable are presented in Table 2. Model 4 of the PROCESS 3.3 macro program was used to analyze the mediating effect of the above variables (Hayes, 2018). The 95% confidence interval (CI) of indirect effects of 5,000 bootstrapped samples was calculated by the percentile bootstrap method. The mediating effect of green attitude was detected as significant between retailer reputation and emotional response [*R*^2^ = 0.445, *F*(2,114) = 45.619, *p* = 0.000]. The total effect of the model was significant, β = 0.585, 95% CI: [0.122, 1.047] (CI did not include 0), *p* = 0.014. However, the direct effect of retailer reputation on emotional response was not significant, β = 0.198, 95% CI: [−0. 167, 0.564] (CI included 0), *p* = 0.284. In accordance with expectations, the indirect effect of the model was significant, 95% CI: [0.085, 0.707] (CI did not include 0). In sum, the findings suggested that the effect of retailer reputation on emotional response was fully mediated by a green attitude (as shown in Table 1).

**TABLE 1:** Results of the mediation analysis with consumption responses as the dependent variables, retailer reputation as the independent variable, and green attitude as the mediator.

Predictors	*B*	95% CI	*P*	*R* ^2^
**Model 1—emotional response as the dependent variable**				
Retailer reputation	0.198	[−0.167, 0.564]	0.284	0.445***
Green attitude	0.711***	[0.554, 0.867]	0.000	
Total effect	0.585*	[0.122, 1.047]	0.014	
Indirect effect	0.386	[0.085, 0.707]		
**Model 2—behavioral response as the dependent variable**				
Retailer reputation	0.729**	[0.302, 1.155]	0.001	0.246***
Green attitude	0.382***	[0.199, 0.565]	0.000	
Total effect	0.936***	[0.494, 1.379]	0.000	
Indirect effect	0.208	[0.030,0.452]		

*p < 0.05, **p < 0.01, ***p < 0.001.

**TABLE 2:** Moderated mediation analysis: Effects of retailer reputation and regulatory focus on consumption response *via* green attitude.

Predictors	*B*	SE	95% CI	*P*	*R*	*R* ^2^	*F*
**Mediating variable: green attitude**							
Constant	0.460*	0.301	[−1.328, −0.136]	0.016	0.360	0.129	5.599**
Retailer reputation	0.460*	0.179	[0.105, 0.814]	0.012			
Regulatory focus	0.785**	0.277	[0.237, 1.333]	0.005			
RR × RF	−0.400*	0.176	[−0.748, −0.052]	0.025			
**Dependent variable: consumption responses**							
Constant	−0.666	0.257	[−1.175, −0.157]	0.11	0.625	0.391	36.604***
Retailer reputation	0.414***	0.153	[0.111, 0.718]	0.008			
Green attitude	0.546***	0.075	[0.397, 0.695]	0.000			

*p < 0.05, **p < 0.01, ***p < 0.001. RR × RF, retailer reputation × regulatory focus.

As was evident from Table 1, retailer reputation was taken as the independent variable, green attitude as the mediating variable, and behavioral response as the dependent variable. Similarly, Model 4 of PROCESS 3.3 was used for the analysis. The mediating effect of green attitude was detected as significant between retailer reputation and behavioral response [*R*^2^ = 0.246, *F*(2,114) = 18.572, *p* = 0.000]. The total effect of the model was significant, β = 0.936, 95% CI: [0.494, 1.379], *p* = 0.000 (CI does not include 0). The direct effect of retailer reputation on behavioral response was significant, β = 0.729, 95% CI: [0.302, 1.155], *p* = 0.001. As expected, the indirect effect of retailer reputation on behavioral response was significant, 95% CI: [0.030, 0.452] (the CI does not include 0). Thus, the effect of retailer reputation on behavioral response was partially mediated by a green attitude.

Mediator analysis was carried out by selecting brand strength as the independent variable, green attitude as the mediating variable, and emotional response as the dependent variable. However, the indirect effect of brand strength on emotional response was insignificant, 95% CI: [−0.051, 0.479] (the CI includes 0), and on behavioral response was also insignificant, 95% CI: [−0.021, 0.294] (the CI includes 0). Thus, the mediating effect was not significant.

**FIGURE 1:**
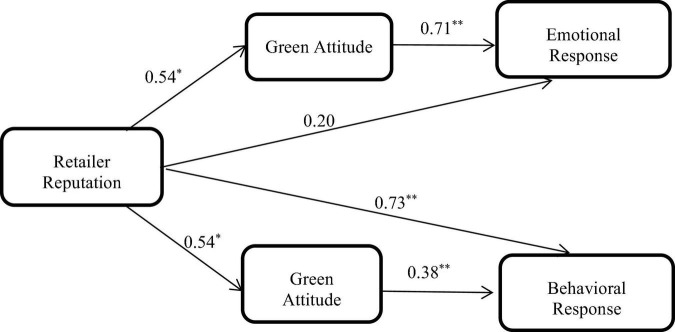
Mediated model for green products with low involvement. *p < 0.05, ^**^p < 0.01.

In sum, the findings suggested that retailer reputation was more important than brand strength in the consumption response of green products with low involvement, and retailer reputation (rather than brand strength) has an important influence on the consumption response mediated by green attitude (presented in Figure 1).

#### The moderated meditation analysis combining retailer reputation and regulatory focus

**FIGURE 2:**
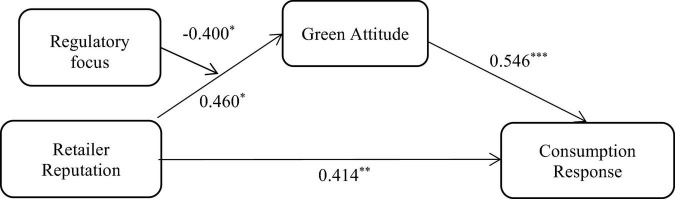
Moderated mediation model for green products with low involvement. *p < 0.05, ^**^p < 0.01, ^***^p < 0.001.

According to the previous literature (Xing et al., 2019), we employed the PROCESS proposed by Hayes (2018) using SPSS 25.0 to test the moderated mediation effect of regulatory focus on the indirect effect of retailer reputation on consumption response *via* green attitude. Then, we specified a moderation mediation model that estimates the indirect effect of X (retailer reputation) on Y (consumption response) *via* M (green attitude) at different levels of V (regulatory focus). Model 7 of the PROCESS was performed. The results showed that the indirect effects of retailer reputation on consumption response *via* green attitude, respectively, were statistically significant when the regulatory focus is low (−1 SD), 95% CI: [0.147, 0.820], and when the regulatory focus equaled the mean, 95% CI: [0.055, 0.476]. However, the indirect effects of retailer reputation on consumption response *via* green attitude were insignificant when the regulatory focus is high (+1 SD), 95% CI: [−0.200, 0.306]. The index of moderated mediation was −0.218, 95% CI: [−0.417, −0.001]. In addition, the results indicate that the indirect effects of retailer reputation on consumption response *via* green attitude decrease (boot effect decreases from 0.469 to 0.033) when the regulatory focus is from low level (−1 SD) to high level (+1 SD), indicating that regulatory focus negatively moderated the indirect effect of retailer reputation on consumption response *via* green attitude (as shown in Table 2). Therefore, our findings revealed that there were moderating effects of regulatory focus on the mediating role of green attitude in the relationship between retailer reputation and consumption response. The model is shown in Figure 2.

## Study 2

### Participants and procedure

A total of 116 undergraduates participated in this study for course credit. The participants were randomly assigned to one of the four cells of a 2 (brand strength: high and low) × 2 (retailer reputation: good and poor) between-subjects design. The manipulations and measurements of the variables were similar to those used in study 1, with the exception of study 2 for green products with high involvement.

### Results

#### Effects of brand strength and retailer reputation on emotional response

Through the analysis of variance, we found that for high-involvement green products, brand strength [*F*(1,112) = 6.190, *p* = 0.014] had significant effects on emotional response. However, retailer reputation [*F*(1,112) = 1.999, *p* = 0.160] had no significant effects on emotional response. The interaction effect between brand strength and retailer reputation was significant [*F*(1,112) = 4.066, *p* = 0.046]. This is consistent with Zhu and Chen (2017), who demonstrated that the interaction was significant between manufacturer and retailer brands on consumer product evaluations. Therefore, we suggest that well-known manufacturers of green high-involvement products should partner with reputable retailers to elicit positive emotional responses.

This study also tested the significance of the moderating analysis by Model 1 of PROCESS. The analysis with selected brand strength as the independent variable, retailer reputation as the moderating variable, and emotional response as the dependent variable is presented in Table 2. The results verified that retailer reputation played a moderating role in the influence of brand strength on emotional response [*R*^2^ = 0.130, *F*(2,114) = 5.563, *p* = 0.001]. The interactive item of brand strength and retailer reputation was significant (β = −1.086, *p* = 0.046), 95% CI: [−2.153, −0.019] (CI does not include 0). According to the hierarchical analysis, it was found that in the low retailer reputation condition, brand strength significantly affected the emotional response (*p* = 0.001, 95% CI: [0.499, 1.928]), and the emotional response of high brand strength was significantly higher than that of low brand strength. It can be seen that brand strength has a great influence on the emotional response of consumers. However, in the high retailer reputation condition, there was no significant effect between brand strength and emotional response (*p* = 0.752, 95% CI: [−0.666, 0.920]). Thus, it can be inferred that when the brand strength was high, there was no strong need for retailer reputation to show signal quality because high brand strength was sufficient. In other words, we speculate that for a product with low brand strength, the benefit of a high retail reputation was greater than that of a product with high brand strength. This was consistent with previous studies showing that a weak manufacturer brand benefits more from a reputable retailer than a strong manufacturer brand (Chu et al., 2005). That is, from the perspective of a manufacturer with low brand strength, it is a beneficial strategy to use the retailer’s good reputation to offset the loss of low brand strength. However, regardless of whether the brand strength was low or high, participants showed a relatively high emotional response in the condition of a high-reputation retailer. It could be seen that it was very important for enterprises to choose a retailer with a high reputation. Consumers might not distinguish the strength of the product brand but tend to choose the retailer they trust. A manufacturer without high brand strength might “borrow” a high retailer reputation to signal quality. Previous studies have shown that a strong retailer brand signals high-quality products and leads to higher consumer purchase intentions (Dodds et al., 1991).

#### The influence of brand strength and retailer reputation on behavioral response

Through the analysis of variance, we found that for low-involvement green products, brand strength [*F*(1,112) = 9.00, *p* = 0.003] and retailer reputation [*F*(1,113) = 8.30, *p* = 0.005] had significant effects on behavior response, but their interaction effect was not significant [*F*(1,113) = 1.36, *p* = 0.25]. The main effect of brand strength was significant [*F*(1,112) = 18.821, *p* = 0.003]. No other effects were significant. The possible reason for this was that the behavioral response was a measure of consumers’ willingness to buy in the future, so the behavioral response was still far away from the time distance. If there was no purchase plan in the near future, consumers were more likely to consider the attributes attached to the product itself, such as the brand, and were unlikely to consider the situational factors of commodity sales, such as retailers.

#### The influence of brand strength, retailer reputation, and regulatory focus on consumption response

The analysis of variance found that the interactions of brand strength, retailer reputation, and regulatory focus on emotional response [*F*(1,108) = 0.894, *p* = 0.346] and behavioral response [*F*(1,108) = 0.537, *p* = 0.465] were not significant. The interaction between brand strength and retailer reputation on emotional response was significant [*F*(1,108) = 4.369, *p* = 0.039], and the effect of brand strength was marginal [*F*(1,108) = 3.870, *p* = 0.052]. In sum, for high-involvement green products, brand strength and retailer reputation played important roles in consumer emotional response. However, the effect of regulatory focus on consumption response was not significant, which was different from the conclusion of study 1 (for low-involvement green products).

Regarding behavioral response, only the main effect of brand strength was significant [*F*(1,108) = 16.911, *p* = 0.000], and the other effects were not significant. For green products with high involvement, whether for consumers who have a promotion focus or prevention focus, it was most important for enterprises to strengthen the construction of the brand image so that consumers could have purchase intention, be loyal to the brand, and then recommend the brand to others.

#### The mediating effect of green attitude between brand strength (vs. retailer reputation) and consumers’ response

Considering the above data analysis, it was found that brand strength played an important role in the consumption response of high-involvement green products. To further explore the internal mechanism, the researchers selected brand strength as an independent variable, green attitude as a mediator variable, and emotional response as a dependent variable by using Model 4 of PROCESS 3.3. The mediating effect of a green attitude was found to be significant between brand strength and emotional response [*R*^2^ = 0.391, *F*(2,113) = 36.206, *p* = 0.000]. The total effect of the model was significant, β = 0.828, 95% CI: [0.303, 1.353] (CI does not include 0), *p* = 0.002. The direct effect of brand strength on emotional response was significant, β = 0.539, 95% CI: [0.103, 0.974], *p* = 0.016. As expected, the indirect effect of the model was significant, 95% CI: [0.010, 0.611] (CI did not include 0). In sum, the findings suggested that the effect of brand strength on emotional response was partially mediated by a green attitude (as shown in Table 2).

Similarly, brand strength was taken as the independent variable, green attitude as the intermediary variable, and behavioral response as the dependent variable by using Model 4 of PROCESS 3.3 for the analysis (as shown in Table 3). The mediating effect of green attitude was detected as significant between brand strength and behavior response [*R*^2^ = 0.364, *F*(2,113) = 32.375, *p* = 0.000]. The total effect of the model was significant, β = 1.111, 95% CI: [0.661, 1.560], *p* = 0.000 (CI does not include 0). The direct effect of brand strength on behavior response was significant, β = 0.906, 95% CI: [0.504, 1.308], *p* = 0.000. As expected, the indirect effect of brand strength on behavior response was significant, 95% CI: [0.009, 0.423] (CI did not include 0). Thus, the effect of brand strength on behavioral response was partially mediated by a green attitude.

**TABLE 3:** Results of the mediation analysis with consumption responses as the dependent variables, brand strength as the independent variable, and green attitude as the mediator.

Predictors	B	95% CI	*P*	*R* ^2^
**Model 1—emotional response as the dependent variable**				
Brand strength	0.539	[0.103, 0.974]	0.016	0.391***
Green attitude	0.624	[0.461, 0.786]	0.000	
Total effect	0.828	[0.303, 1.353]	0.002	
Indirect effect	0.290	[0.010, 0.611]		
**Model 2—behavioral response as the dependent variable**				
Brand strength	0.906	[0.504, 1.308]	0.000	0.364***
Green attitude	0.441	[0.291, 0.591]	0.000	
Total effect	1.111	[0.661, 1.560]	0.000	
Indirect effect	0.205	[0.009, 0.423]		

*p < 0.05, **p < 0.01, ***p < 0.001.

Finally, mediator analysis was carried out by selecting retailer reputation as the independent variable, green attitude as the mediator variable, and emotional response and behavioral response as the dependent variables. However, the indirect effect of retailer reputation on emotional response was insignificant, 95% CI: [−0.058, 0.543] (the CI includes 0), and on behavioral response was also insignificant, 95% CI: [−0.027, 0.314] (the CI includes 0). Thus, the results showed that the indirect effects were not significant.

**FIGURE 3:**
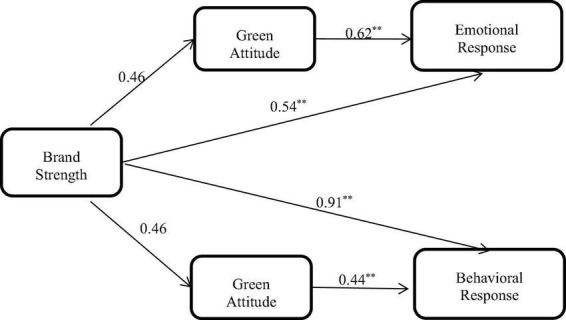
Mediated model for green products with high involvement. In estimating the model, we tested a mediated effect. ^**^p < 0.01.

As shown in Figure 3, among the factors affecting the consumption of green products with high involvement, brand strength was more important than retailer reputation. Brand strength (rather than retailer reputation) had an important impact on consumer response mediated by green attitude.

#### The moderated meditation analysis combining brand strength and regulatory focus

Similarly, we employed the PROCESS proposed by Hayes (2018) to test the moderated mediation effect of regulatory focus on the indirect effect of brand strength on consumption response *via* green attitude. Then, we specified a moderation mediation model that estimates the indirect effect of X (brand strength) on Y (consumption response) *via* M (green attitude) at different levels of V (regulatory focus). Model 7 of the PROCESS was performed. The results showed that the indirect effects of brand strength on consumption response *via* green attitude were insignificant when the regulatory focus was low (−1 SD), 95% CI: [−0.048, 0.478]. The indirect effects of brand strength on consumption response *via* green attitude were insignificant when the regulatory focus was high (+1 SD), 95% CI: [−0.089, 0.494]. The index of moderated mediation was −0.008, 95% CI: [−0.207, 0.192]. Therefore, the findings revealed that there were no moderating effects of regulatory focus on the mediating role of green attitude in the relationship between brand strength and consumption response.

## Data summary analysis

After the evaluation and analysis of the above two studies, one group of materials about low-involvement green products (117 respondents) and the other group of materials about high-involvement green products (116 respondents) were aggregated to form a single base of 233 observations.

First, we conducted a mediation analysis (Model 4) with green attitudes as the mediator. The results of a mediation analysis suggested that retailer reputation (effect: 0.210, SE = 0.078, 95% CI: [0.061, 0.367]) or brand strength (effect: 0.181, SE = 0.077, 95% CI: [0.036, 0.336]) could significantly promote green attitude, which in turn led to positive consumption response.

**FIGURE 4:**
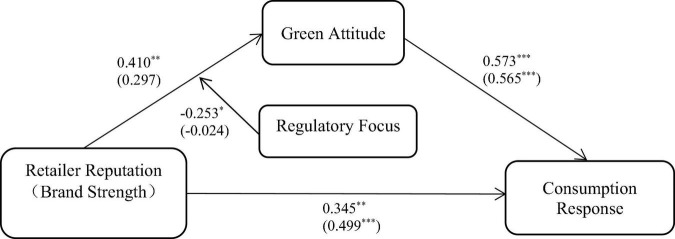
Moderated mediation model for low- and high-involvement green products. The numbers in brackets are the path coefficients of the model [green attitude (M) as a mediator and regulatory focus (W) as a moderator of the effect of brand strength (X) on consumption response (Y)]. *p < 0.05, ^**^p < 0.01, ***p < 0.001.

Next, we tested green attitude (M) and regulatory focus (W) as a moderator of the effect of retailer reputation (X) on consumption response (Y). Model 7 of the PROCESS was performed. Figure 4 presents the results of the moderated mediation analysis. The results indicated that the interaction effect of retailer reputation and regulatory focus on green attitude was significant (β = −0.253, 95% CI: [−0.504, −0.002]). The index of moderated mediation for the conditional indirect effect of retailer reputation on consumption response through green attitude was significant (index = −0.145, 95% CI: [−0.288, −0.002]). When the regulatory focus is low (*M* − 1 SD), the mediating effect of green attitude on the relationship between retailer reputation and consumption response was significant (effect: 0.379, 95% CI: [0.164, 0.621]). Additionally, when the regulatory focus is high (*M* + 1 SD), the mediating effect of green attitude on the relationship between retailer reputation and consumption response was insignificant (effect: 0.090, 95% CI: [−0.094, 0.290]). As expected, the direct effect of retailer reputation on consumption response was significant, β = 0.345, 95% CI: [0.140, 0.550], *p* = 0.001, and the effect of green attitude on consumption response was also significant, β = 0.573, 95% CI: [0.470, 0.676], *p* < 0.001. Similar to the above, we tested green attitude and regulatory focus as a mediator and a moderator of the relationship between brand strength and consumption response. However, we did not find statistically significant moderation effects (Figure 4). The results indicated that the interaction effect of brand strength and regulatory focus on green attitude was insignificant (β = −0.024, 95% CI: [−0.279, 0.231]). The index of moderated mediation for the conditional indirect effect of brand strength on consumption response through green attitude was also insignificant (index = −0.013, 95% CI: [−0.163, 0.142]). However, the direct effect of brand strength on consumption response was significant, β = 0.499, 95% CI: [0.300, 0.697], *p* < 0.001, and the effect of green attitude on consumption response was also significant, β = 0.565, 95% CI: [0.465, 0.664], *p* < 0.001.

Finally, we explored whether the product involvement has a moderated mediating effect in the model of Figure 4 by using Model 9 from PROCESS. As expected, the results revealed the direct effect of retailer reputation or brand strength on consumption response was significant. However, the indirect effect of retailer reputation on consumption response through green attitude was not moderated by product involvement (β = −0.088, SE = 0.261, 95% CI: [−0.602, 0.425]), and the indirect effect of brand strength was not also moderated by product involvement (β = 0.041, SE = 0.263, 95% CI: [−0.477, 0.558]).

## Discussion

With the rapid development of the social economy, people’s consumption structure has been continuously optimized, and the consumption level has been constantly improved. At the same time, the environmental pollution and damage caused by excessive consumption of resources by economic development are becoming increasingly significant. Global society has reached a consensus that green consumption will contribute to sustainable development. In recent years, there have been an increasing number of studies on green consumption, which have important theoretical and practical significance for understanding green consumption. The current article explores how product clues (brand strength and retailer reputation) affect consumption responses to green products, and to examine the mediating effect of green attitude and the moderating effect of regulatory focus, through two studies on the types of products with low and high involvement. This research has the following three main findings.

First, this research explores the influence mechanism of consumption response to low-involvement products and high-involvement products, respectively. The results of study 1 reveal that for low-involvement green products, the effect of the retailer reputation rather than brand strength on consumption response is mediated by a green attitude. It can be seen that for green products with low involvement, retail reputation plays a more prominent role than brand strength in shaping green attitudes and thus promoting consumer response. For low-involvement products, consumers are likely to be influenced by advertisements and promotions, but it is not easy to form customers’ brand loyalty. Consumers are more perceptual in the process of buying low-involvement products and have less interest in product information (Morgan-Thomas and Veloutsou, 2013). Therefore, consumers invest less time and energy in comparing and analyzing low-involvement products. Instead, retailer reputation and advertising have become important sources of information for consumers to buy products. In addition, online retailer reputation is more represented by numbers and scores, which is easier to process and can be regarded as a heuristic cue. Therefore, when facing low-involvement products, consumers’ motivation to deal with complex product information may be reduced, and they rely more on simple and credible retailer reputation information (Wang et al., 2016), while the results of study 2 show that for high-involvement green products, the effect of the brand strength rather than retailer reputation on consumption response is mediated by a green attitude. It can be seen that for green products with high involvement, brand strength plays a more prominent role than retail reputation in shaping green attitudes and thus promoting consumer response. In the case of highly involved products, consumers are more inclined to rational judgment, spend a lot of time on product selection and comparison, and pay more attention to the professionalism and credibility of information. In other words, high involvement will increase the level of interest in related stimuli, as well as the intensity and depth of information processing (Mittal, 1995). With this depth information treatment, consumers have strong perceptions of the ability of the enterprise, and they will strive to increase brand awareness to more accurately evaluate the product quality of the enterprise (Petty et al., 1983). This was also argued by Mühlbacher et al. (2016), who stated that product involvement had an impact on the correlation feature patterns that lead to high or low brand strength. Our results also show that brand strength plays a key role in the consumption decision of high-involvement products, which is consistent with prior research (Yang and Hu, 2022). As can be seen from the above, it is essential for merchants to understand consumers’ evaluation and attitude toward the high-range clues, such as brand strength for high-involvement products and retailer reputation for low-involvement products. With the development of artificial intelligence service technology, customers’ brand perception and satisfaction can be carried out according to machine learning algorithms and big data (Kliestik et al., 2022). In recent years, many scholars are discussing how online purchase decision algorithm and self-service technology have changed consumption and purchasing habits in the retail environment. Scholars have found that consumers’ purchase behavior and decision-making routines can be assisted by machine and deep learning algorithms, system data processing, and big data analysis. These technologies have improved users’ lifestyle and payment methods, enhanced customer experience, and then improved consumer satisfaction (Hopkins, 2022; Kliestik et al., 2022). With the increasing evidence of online purchase decision algorithm, how to use high-tech services to improve the utility of product clues has gradually become an emerging topic involving much interest.

Second, the two studies show that whether for high-involvement or low-involvement products, a green attitude plays an important intermediary role in green consumption response. In research on the influencing factors of green consumption, many scholars believe that environmental attitude is an important influencing factor of green consumption. Because the green brand attitude will indirectly influence green purchase intention, while companies tend to raise their customers’ green purchase intentions, they need to increase their green attitudes (Chen et al., 2020). Some studies have also shown that consumer green attitude mediates the influence of green product knowledge, green product orientation, and social influence on behavioral intention (Chen et al., 2022). Green attitude has a significant impact on green product consumption behavior, and consumer attitude is an important predictor of consumers’ green product consumption behavior intention (Ogiemwonyi and Harun, 2020). Therefore, the government and relevant departments should jointly carry out targeted publicity and environmental education, such as emphasizing the seriousness of environmental problems and advocating environmental protection actions. It is necessary to formulate effective environmental protection policies to effectively improve the level of consumers’ green attitude, which is conducive to promoting the response of green consumption.

Finally, for the green products with low involvement, the moderating role of regulatory focus in the indirect effect of retailer reputation on consumption responses *via* green attitude is confirmed. However, for the green products with high involvement, the moderating role of regulatory focus in the indirect effect of brand strength on consumption responses is insignificant *via* green attitude. Previous studies have found that individuals with different regulatory foci value product cues differently. Specifically, consumers with a promotion focus think that external cues are more important, while consumers with a prevention focus think that internal cues are more important (Song and Morton, 2016). However, this may ignore product involvement. The role of regulatory focus depends on the level of involvement in activities (Avnet et al., 2013). For products with high involvement, consumers would have persistent interests and concerns about products and engage in searching and processing information (Bloch, 1981). That is, as involvement increases, consumers are more motivated to allocate limited cognitive resources to process information (Petty and Cacioppo, 1990). In the decision-making process of high-involvement products, consumers would comprehensively search and carefully process product clues, and the moderating effect of moderating regulatory focus does not exist (Li and Yu, 2021). This is confirmed in our study 2. Therefore, practitioners should consider various characteristics of product categories, including involvement, to achieve marketing objectives. In addition, previous studies have mostly concerned that regulatory focus works as the antecedent to consumers’ evaluation of products or services and plays independent and interactive roles in the field of marketing and retailing researches (Chang et al., 2019; Tran et al., 2020). Hence, the related researches should not only pay attention to the effects of regulatory focus as the antecedent, but also pay attention to the moderating effects of regulatory focus on other influence paths.

## Conclusion

In this study, an experimental method is used to explore the influence mechanism of consumption response to green products. The results show that for low-involvement green products, the reputation of retailers is an important predictive variable in consumption response mediated by green attitude. However, for green products with high involvement, brand strength is a key factor affecting consumers’ response mediated by a green attitude. In addition, our study also found that regulatory focus has (does not have) a certain effect on the consumption response of low- (high-) involvement green products.

### Theoretical implications

By applying the cue-diagnosticity theory, the main theoretical implication of the study involves the effectiveness of high-range clues (brand strength and retailer reputation) in the decision-making process of purchasing of products with different involvement. Previous research results were cumulated showing that brand and retailer reputation are high-range clues that consumers attach importance to. In this study, we show that the influence mechanism of brand strength and retailer reputation on products with different involvement might be different, so as to grasp the consumer journey more detailed and in depth, thus improving the clue diagnosis theory.

Other theoretical implication of the manuscript involves the need to explore the green attitude as a mediator and regulatory focus as a moderator in the relationship between the high-range clues (brand and retailer reputation) and consumption response, something that is clearly unprecedented in the literature. The green attitude plays a mediator effect in the relationship between brand strength (for high-involvement products) or retailer reputation (low-involvement products) and consumer response. It can be noted that green attitude exerts an important role in consumption response. Additionally, regulatory focus plays a moderator role in the consumption response of low-involvement products rather than high-involvement products, again highlighting and deepening the differences in the internal mechanism of triggering consumption response.

Finally, the mechanism of influencing consumption decision in the green market is different from that in the non-green market. This research expands the application scope of the clue diagnosticity theory. Therefore, the cue-diagnosticity theory requires refinement in order to be used to evaluate the relationship between different types of product clues and consumption responses in the green market.

### Managerial contributions

The findings of this study reveal some strategic approaches and will provide decision-makers and marketing managers with valuable insights into the key determinants of green consumption response. For the marketing or production of low-involvement green products, it is very important to choose a reputable retailer; in addition, in specific marketing scenarios, some information frameworks should start different regulatory focuses to promote the generation of green consumption response. For example, for laundry detergent (low involvement), even though the brand strength of the green product is not high, if the enterprise chooses a retailer with a good reputation, it is likely to get a better consumer response, especially for those consumers with prevention focus. If the brand strength of laundry detergent is not high and the retailer reputation is not good enough, marketers can initiate a temporary promotion state of consumers through some marketing strategies, such as presenting some gain-framed messages to consumers, which may promote their positive consumption response. However, methods that may be effective for one green product may not work for another. For highly involved products, consumers pay more attention to the product brand and tend to invest more time and energy in understanding the product. Because the brand image is an integral part of value, the greater the brand strength, the greater the perceived value and credibility of consumers. For example, for green cars (high involvement), the brand strength of cars is an important predictor of consumer response, whether for consumers with promotion focus or prevention focus. If manufacturers can produce better performing and more cost-effective green cars, that is, with high brand strength, then they have a good chance to find more and more consumer acceptance. Therefore, enterprises and departments related to green products should strengthen the evaluation and management of the degree of consumer involvement and implement corresponding marketing strategies according to the degree of involvement. Moreover, the findings highlight the significant mediating role of green attitude toward consumption response. This implies that higher green product attitude may increase higher green product consumption response. Therefore, the government and enterprises should jointly advocate environmental protection actions, strengthen the publicity of green product, and formulate effective environmental protection policies to improve consumers’ green attitude. That is to say, relevant personnel should set as an important goal to create and improve consumers’ green attitude. Marketers should not only pay attention to the value of product clues, but also focus on the green attitudes of consumers.

### Limitations and future research perspectives

This study provides references for some environmental protection enterprises and other application fields. However, there are still some deficiencies in the current research. First, the subjects in this study were college students. Although all the experimental designs adopt the intergroup approach to avoid contamination, there are great differences among consumers at all levels between college students and different age groups. Therefore, there are some differences between the experimental subjects and ordinary consumers, which need to be verified and promoted by a large number of other subjects. In addition, the experimental material used in this study is virtually written material composed of pictures and words to verify the hypotheses, which is different from the real consumption environment and is limited by the restrictions of the research conditions and the quality of the experimental material itself. This may affect the final research conclusion, so it is suggested that researchers should use realistic materials to verify the research conclusion. Third, our research explores the factors that affect the response of green consumption in the context of the Chinese culture. However, there are important differences in ethical behavior in different cultures because individuals’ cognition and interpretation of morality are influenced by culture. Although consumers have a positive attitude toward green consumption in the Chinese culture, because green consumption is still a relative minority, consumers are more likely to cater to social norms rather than inherent green consciousness in decision-making. In addition, green products are new and have not been fully tested by the market. Chinese consumers’ high uncertainty aversion will eventually drive consumers to choose mainstream products rather than new green products in their actual purchase decisions. Therefore, the cross-cultural comparison of green consumption should be given more attention.

## Data availability statement

The original contributions presented in this study are included in the article/supplementary material, further inquiries can be directed to the corresponding author.

## Ethics statement

Ethical review and approval was not required for the study on human participants in accordance with the local legislation and institutional requirements. The patients/participants provided their written informed consent to participate in this study.

## Author contributions

XW: conceptualization, project administration, writing—review and editing, and methodology. YG: funding acquisition and resources. HX: investigation and writing—original draft preparation. PQ: data curation and software. JW: supervision, project administration, and visualization. All authors have read and agreed to the published version of the manuscript.

## Appendix A

**APPENDIX TABLE A1:** The questionnaires.

Scales	Items		References
	Numbers	Content	
Brand strength	A1	What do you think about the brand strength of the brand?	Xiangdong, 2017
	A2	What is the status of this brand in the industry?	
Retailer reputation	B1	The retailer’s score (description, service, and delivery) is above the industry average	Purohit and Srivastava, 2001; Chao, 2016
	B2	The retailer has a high cumulative sales and reviews	
	B3	The retailer has a high overall reputation score	
Green attitude	C1	I prefer green products because they satisfy my values	Al-Swidi and Saleh, 2021
	C2	I prefer green products because it is environment-friendly	
	C3	I believe that green products are competitive	
	C4	It is exciting for me to buy green products	
Regulatory focus	D1	I have been striving to fulfill my hopes and aspirations	Higgins, 1997; Bai-Lin, 2015
	D2	I am more concerned with getting positive results in my life	
	D3	I work hard in pursuit of success and progress	
	D4	I am more concerned about the positive outcomes of things and strive for them	
	E5	I am always anxious that I will fall short of my responsibilities and obligations	
	E6	I am more focused on avoiding negative outcomes in my life	
	E7	I work hard to prevent failure and falling behind	
	E8	I am more concerned about the potential threats or negative incidents and pay attention to avoid them	
Emotional response	F1	I am eager to learn more information of this brand related to environmental protection	Yong, 2020
	F2	I am willing to pay a higher price for products from environment-friendly brands	
	F3	I am willing to participate in related environmental protection activities by purchasing this brand product.	
Behavior response	G1	I will continue buying products from this brand	
	G2	I will be loyal to the brand	
	G3	I will recommend the brand to others	

## References

[B1] Akdeniz B. Calantone R. J. Voorhees C. M. (2013). Effectiveness of marketing cues on consumer perceptions of quality: The moderating roles of brand reputation and third-party information. *Psychol. Mark.* 30 76–89. 10.1002/mar.20590

[B2] Al-Swidi A. Saleh R. M. (2021). How green our future would be? An investigation of the determinants of green purchasing behavior of young citizens in a developing Country. *Environ. Dev. Sustain.* 23 13436–13468. 10.1007/s10668-020-01220-z

[B3] Avnet T. Higgins E. T. (2006). How regulatory fit affects value in consumer choices and opinions. *J. Mark. Res.* 43 1–10. 10.1509/jmkr.43.1.1 11670861

[B4] Avnet T. Lauferr D. Higgins E. T. (2013). Are all experiences of fit created equal? Two paths to persuasion. *J. Consum. Psychol.* 23 301–316. 10.1016/j.jcps.2012.10.011

[B5] Bai-Lin C. (2015). A study on the effect of WOM on social media: Focus on construal level theory and regulatory focus theory. *Jinan J. Philos. Soc. Sci. Ed.* 37 99–105+163.

[B6] Bloch P. H. (1981). An exploration into the scaling of consumers’ involvement with a product class. *Adv. Consum. Res.* 8:61.

[B7] Cesario J. Grant H. Higgins E. T. (2004). Regulatory fit and persuasion: Transfer from “Feeling Right”. *J Pers Soc Psychol.* 86 388–404. 10.1037/0022-3514.86.3.388 15008644

[B8] Chang K. Hsu C. Hsu Y. Chen M. (2019). How green marketing, perceived motives and incentives influence behavioral intentions. *J. Retail. Consum. Serv.* 49 336–345. 10.1016/j.jretconser.2019.04.012

[B9] Chao M. (2016). *Examining the effects of multi-cues on consumers’ product evaluation: A cognitive cue framework.* Zhejiang: Zhejiang University.

[B10] Chen X. Rahman M. K. Rana M. S. Gazi M. A. I. Rahaman M. A. Nawi N. C. (2022). Predicting consumer green product purchase attitudes and behavioral intention during Covid-19 pandemic. *Front. Psychol.* 12:760051. 10.3389/fpsyg.2021.760051 35145450 PMC8822218

[B11] Chen Y. Chang T. Li H. Chen Y. (2020). The influence of green brand affect on green purchase intentions: The mediation effects of green brand associations and green brand attitude. *Int. J. Environ. Res. Public Health* 17:4089. 10.3390/ijerph17114089 32521728 PMC7311963

[B12] Cheung M. F. Y. To W. M. (2019). An extended model of value-attitude-behavior to explain Chinese consumers’ green purchase behavior. *J. Retail. Consum. Serv.* 50 145–153. 10.1016/j.jretconser.2019.04.006

[B13] Chu W. Chu W. (1994). Signaling quality by selling through a reputable retailer: An example of renting the reputation of another agent. *Mark. Sci.* 13 177–189. 10.1287/mksc.13.2.177 19642375

[B14] Chu W. Choi B. Song M. R. (2005). The role of on-line retailer brand and infomediary reputation in increasing consumer purchase intention. *Int. J. Electron. Commer.* 9 115–127. 10.1080/10864415.2005.11044336

[B15] Cox D. F. (1962). “The measurement of information value: A study in consumer decision-making,” in *Emerging concept in marketing*, ed. Decker W. S. (Chicago, IL: American Marketing Association), 413–421.

[B16] Dǎbija D. Babut R. (2019). Enhancing apparel store patronage through retailers’ attributes and sustainability. A generational approach. *Sustainability* 11:4532. 10.3390/su11174532

[B17] Dabija D. Bejan B. M. Grant D. B. (2018). The impact of consumer green behaviour on green loyalty among retail formats: A romanian case study. *Morav. Geogr. Rep.* 26 173–185. 10.2478/mgr-2018-0014

[B18] Dhir A. Sadiq M. Talwar S. Sakashita M. Kaur P. (2021). Why do retail consumers buy green apparel? A knowledge-attitude-behaviour-context perspective. *J. Retail. Consum. Serv.* 59:102398. 10.1016/j.jretconser.2020.102398

[B19] Dodds W. B. Monroe K. B. Grewal D. (1991). Effects of price, brand, and store information on buyers’ product evaluations. *J. Mark. Res.* 28 307–319. 10.1177/002224379102800305

[B20] Erdem T. Swait J. Valenzuela A. (2006). Brands as signals: A cross-country validation study. *J. Mark.* 70 34–49. 10.1509/jmkg.70.1.034.qxd 11670861

[B21] Gidron D. Koehler D. J. Tversky A. (1993). Implicit quantification of personality traits. *Pers. Soc. Psychol. Bull.* 19 594–604. 10.1177/0146167293195011

[B22] Grewal D. Monroe K. B. Krishnan R. (1998). The effects of price-comparison advertising on buyers’ perceptions of acquisition value, transaction value, and behavioral intentions. *J. Mark.* 62 46–59. 10.1177/002224299806200204

[B23] Han H. Kim Y. (2010). An investigation of green hotel customers’ decision formation: Developing an extended model of the theory of planned behavior. *Int. J. Hosp. Manag.* 29 659–668. 10.1016/j.ijhm.2010.01.001

[B24] Hayes A. F. (2018). *Introduction to mediation, moderation, and conditional process analysis: A regression-based approach.* New York, NY: Guilford Press.

[B25] Higgins E. T. (1997). Beyond pleasure and pain. *Am. Psychol.* 52 1280–1300. 10.1037/0003-066X.52.12.1280 9414606

[B26] Hopkins E. (2022). Machine learning tools, algorithms, and techniques in retail business operations: Consumer perceptions, expectations, and habits. *J. Self Gov. Manag. Econ.* 10 43–55. 10.22381/jsme1012023

[B27] Jian M. Wenlong Z. Morad B. (2020). Impact of product description and involvement on purchase intention in cross-border e-commerce. *Ind. Manag. Data Syst.* 120 567–586. 10.1108/IMDS-05-2019-0280

[B28] Kim Y. J. Han J. (2014). Why smartphone advertising attracts customers: A model of Web advertising, flow, and personalization. *Comput. Hum. Behav.* 33 256–269. 10.1016/j.chb.2014.01.015

[B29] Kliestik T. Zvarikova K. Lazaroiu G. (2022). Data-driven machine learning and neural network algorithms in the retailing environment: Consumer engagement, experience, and purchase behaviors. *Econ. Manag. Financial Mark.* 17 57–69. 10.22381/emfm17120224

[B30] Korfiatis N. García-Bariocanal E. Sánchez-Alonso S. (2012). Evaluating content quality and helpfulness of online product reviews: The interplay of review helpfulness vs. review content. *Electron. Commer. Res. Appl.* 11 205–217. 10.1016/j.elerap.2011.10.003

[B31] Kumar B. Manrai A. K. Manrai L. A. (2017). Purchasing behaviour for environmentally sustainable products: A conceptual framework and empirical study. *J. Retail. Consum. Serv.* 34 1–9. 10.1016/j.jretconser.2016.09.004

[B32] Lǎzǎroiu G. Andronie M. Utǎ C. Hurloiu I. (2019). Trust management in organic agriculture: Sustainable consumption behavior, environmentally conscious purchase intention, and healthy food choices. *Front. Public Health* 7:340. 10.3389/fpubh.2019.00340 31803705 PMC6877712

[B33] Li D. Yu W. (2021). Research on the formation mechanism of consumers’ green purchase intention: A role of advertising goal framing and regulatory focus. *J. Nanjing Univ. Technol. Soc. Sci. Ed.* 20 87–98.

[B34] Meng L. Jiao T. X. Liu F. J. (2019). The influence of internet language advertising and regulatory focus on consumers purchase intention. *China Bus. Mark.* 33 98–108. 10.14089/j.cnki.cn11-3664/f.2019.06.010

[B35] Mittal B. (1995). A comparative analysis of four scales of consumer involvement. *Psychol. Mark.* 12 663–682. 10.1002/mar.4220120708

[B36] Morgan-Thomas A. Veloutsou C. (2013). Beyond technology acceptance: Brand relationships and online brand experience. *J. Bus. Res.* 66 21–27. 10.1016/j.jbusres.2011.07.019

[B37] Mühlbacher H. Raies K. Grohs R. Koll O. (2016). Drivers of brand strength: Configural paths to strong cognitive brand equity. *J. Bus. Res.* 69 2774–2780. 10.1016/j.jbusres.2015.11.013

[B38] Ogiemwonyi O. Harun A. B. (2020). Consumption of green product as a means of expressing green behaviour in an emerging economy: With the case study of Malaysia. *Environ. Urban. ASIA* 11 297–312. 10.1177/0975425320938538

[B39] Olson E. L. (2013). It’s not easy being green: The effects of attribute tradeoffs on green product preference and choice. *J. Acad. Mark. Sci.* 41 171–184. 10.1007/s11747-012-0305-6

[B40] Olson J. C. Jacoby J. (1972). “Cue utilization in the quality perception process,” in *Proceedings of the 3rd annual conference of the association for consumer research*, ed. Venkatesan M. (Chicago, IL: Association for Consumer Research), 167–179.

[B41] Paul J. Modi A. Patel J. (2016). Predicting green product consumption using theory of planned behavior and reasoned action. *J. Retail. Consum.Serv.* 29 123–134. 10.1016/j.jretconser.2015.11.006

[B42] Petty R. E. Cacioppo J. T. (1990). Involvement and persuasion: Tradition versus integration. *Psychol. Bull.* 107 367–374. 10.1037/0033-2909.107.3.367

[B43] Petty R. E. Cacioppo J. T. Schumann D. (1983). Central and peripheral routes to advertising effectiveness: The moderating role of involvement. *J. Consum. Res.* 10 135–146. 10.1086/208954

[B44] Purohit D. Srivastava J. (2001). Effect of manufacturer reputation, retailer reputation, and product warranty on consumer judgments of product quality: A cue diagnosticity framework. *J. Consum. Psychol.* 10 123–134. 10.1207/s15327663jcp1003_1

[B45] Rahman I. (2018). The interplay of product involvement and sustainable consumption: An empirical analysis of behavioral intentions related to green hotels, organic wines and green cars. *Sustain. Dev.* 26 399–414. 10.1002/sd.1713

[B46] Rao A. R. Monroe K. B. (1988). Moderating effect of prior knowledge on cue utilization in product evaluations. *J. Consum. Res.* 15 253–264. 10.1086/209162

[B47] Riskos K. Dekoulou P. Mylonas N. Tsourvakas G. (2021). Ecolabels and the attitude–behavior relationship towards green product purchase: A multiple mediation model. *Sustainability* 13:6867. 10.3390/su13126867

[B48] Rothschild M. L. (1984). Perspectives on involvement: Current problems and future directions. *Adv. Consum. Res.* 11 216–217.

[B49] Scalco A. Noventa S. Sartori R. Ceschi A. (2017). Predicting organic food consumption: A meta-analytic structural equation model based on the theory of planned behavior. *Appetite* 112 235–248. 10.1016/j.appet.2017.02.007 28188865

[B50] Solomon M. R. (2011). *Consumer behavior: Buying, having, and being*, 9th Global Edn. Boston, MA: Pearson.

[B51] Song D. Morton C. R. (2016). The influence of regulatory focus on the effect of product cues. *Psychol. Mark.* 33 917–933. 10.1002/mar.20928

[B52] Tran T. P. Guzmán F. Paswan A. K. Blankson C. (2020). National versus private brand: A regulatory focus perspective. *J. Retail. Consum. Serv.* 57:102198. 10.1016/j.jretconser.2020.102198

[B53] Vakratsas D. Ambler T. (1999). How advertising works: What do we really know? *J. Mark.* 63 26–43. 10.1177/002224299906300103

[B54] Vijayasarathy L. R. Jones J. M. (2015). “Perceptions of internet shopping: An experimental comparison of new entrant and established catalogers,” in *Proceedings of the 1999 Academy of Marketing Science (AMS) annual conference*, ed. Noble C. (Cham: Springer). 10.1007/978-3-319-13078-1_12

[B55] Wang C. Li Y. Luo X. Fu H. Ye Z. Deng G. (2022). How are consumers affected by taste and hygiene ratings when ordering food online? A behavioral and event-related potential study. *Front. Neurosci.* 16:844027. 10.3389/fnins.2022.844027 35386593 PMC8978544

[B56] Wang Q. Cui X. Huang L. Dai Y. (2016). Seller reputation or product presentation? An empirical investigation from cue utilization perspective. *Int. J. Inf. Manag.* 36 271–283. 10.1016/j.ijinfomgt.2015.12.006

[B57] Wang Y. M. Zaman H. M. F. Alvi A. K. (2022). Linkage of green brand positioning and green customer value with green purchase intention: The mediating and moderating role of attitude toward green brand and green trust. *SAGE Open* 12:21582440221. 10.1177/21582440221102441

[B58] Xiangdong J. (2017). Spillover effects of strong brands competition. *Acta Psychol. Sin.* 50 678–692. 10.3724/SP.J.1041.2018.00678

[B59] Xing X. Wang J. Tou L. (2019). The relationship between green organization identity and corporate environmental performance: The mediating role of sustainability exploration and exploitation innovation. *Int. J. Environ. Res. Public Health* 16:921. 10.3390/ijerph16060921 30875787 PMC6466600

[B60] Xuemei B. Luiz M. (2011). The role of brand image, product involvement, and knowledge in explaining consumer purchase behaviour of counterfeits: Direct and indirect effects. *Eur. J. Mark.* 45 191–216. 10.1108/03090561111095658

[B61] Yang C. Hu J. (2022). When do consumers prefer AI-enabled customer service? The interaction effect of brand personality and service provision type on brand attitudes and purchase intentions. *J. Brand Manag.* 29 167–189. 10.1057/s41262-021-00261-7

[B62] Yong Z. (2020). *Research on the effect of consumer perception towards green marketing on consumer response.* Liaoning: Liaoning University.

[B63] Zhu Y. Chen H. A. (2017). A tale of two brands: The joint effect of manufacturer and retailer brands on consumers’ product evaluation. *J. Brand Manag.* 24 284–306. 10.1057/s41262-017-0034-8

